# An approach to targeting Nav1.7 for pain sensations

**DOI:** 10.1172/JCI194126

**Published:** 2025-07-15

**Authors:** Theodore R. Cummins

**Affiliations:** Department of Biology, School of Science and Stark Neurosciences Research Institute, Indiana University, Indianapolis, Indiana, USA.

## Abstract

Pain is a serious medical condition with current treatments remaining limited by side effects. The Nav1.7 voltage-gated sodium channel is a crucial determinant of nociceptor excitability and a promising target for nonaddictive analgesics. However, development of blockers has been difficult. In this issue of the *JCI*, Singh, Bernabucci, and authors identify a strategy for reducing Nav1.7 currents. These findings identify fibroblast growth factor 13 (FGF13), a homologous factor distinct from typical growth factors (also known as FHF2B), which ramps up Nav1.7, nociceptor excitability, and pain. Compound PW164 was identified as a selective FGF13-Nav1.7 attenuator with analgesic activity. These findings highlight the power of targeting intrinsic modulators of Nav1.7 for pain management.

## Targeting pain by reducing nociceptor sodium currents

Pain treatment is an important medical challenge. Although approved drugs often effectively reduce pain, these are limited by side effects, including cardiac actions and addiction (1). Tremendous effort has been exerted to identify novel strategies to treat pain. One promising target has been voltage-gated sodium channels (VGSCs) in nociceptive neurons (2). Indeed, recently a Nav1.8 selective inhibitor was approved for the treatment of pain (3). However, there is also substantial interest in developing strategies that target Nav1.7 (4).

In excitable cells, VGSCs generate the upstroke of action potentials and contribute to subthreshold activity (2). The nine sodium channel genes have tissue-specific expression, and selective targeting can affect excitability associated with specific physiological modalities, especially in the instance of pain. Because Nav1.7, Nav1.8, and Nav1.9 are the predominant VGSC isoforms expressed in nociceptive neurons, targeting these subtypes individually or in combination should provide for powerful analgesia (5). Specifically, Nav1.7 contributes to subthreshold and threshold currents in nociceptive neurons (6). Individuals with biallelic loss-of-function variants in Nav1.7 exhibit a pronounced lack of pain sensations. While these individuals also experience anosmia, they otherwise have normal physiological functioning. Conversely, gain-of-function variants that enhance Nav1.7 activation — and/or impair Nav1.7 inactivation gating — lead to severe inherited pain syndromes. These genetic findings along with neurophysiological findings validate that Nav1.7 is a major determinant of nociception and driver of pain sensations (4, 6). While opioids, the gold standard for pain medication, exhibit substantial problems due to addiction, tolerance, and dependence associated with their use (1), there has been hope that selective Nav1.7 blockers could provide pronounced analgesia without addiction due to the restricted expression of Nav1.7 in the peripheral nervous system (6, 7). Several Nav1.7 selective blockers have been developed; however, because Nav1.7 is expressed in both the somatosensory nervous system and the sympathetic nervous system, these potential analgesic drugs can be associated with autonomic dysfunction (8).

## Targeting intrinsic modulators as an alternative approach

Alternative approaches, such as targeting intrinsic modulators of Nav1.7 currents in nociceptive neurons, have recently been investigated in the hopes of developing Nav1.7 inhibitors with fewer side effects (9). An intriguing study using a transgenic mouse with an epitope-tagged Nav1.7 indicated that as many as 267 proteins are associated with Nav1.7 in vivo (10). In this issue of the *JCI*, Singh, Bernabucci, and authors targeted a well-documented Nav1.7 interactor, FGF13, with promising results (11).

FGF13 belongs to a group of proteins produced by a subset of genes in the fibroblast growth factor family, FGF11 through FGF14. These factors have emerged as major modulators of VGSCs (12). The proteins are also known as fibroblast growth factor homologous factors (FHF1 through FHF4) because, unlike other FGFs, they do not bind FGF receptors and are not secreted like classic FGFs. The FHF terminology is generally used to distinguish these intracellular proteins from the classic FGFs. A sequence of approximately 125 amino acids at the core of these FHFs adopts a β-trefoil structure similar to that of true FGFs but with only about 30% sequence identity (13). FHF core regions have been shown to bind to the c-terminus of VGSCs, just upstream of where calmodulin binds to VGSCs (14). Intriguingly, there are multiple FHF isoforms, with each FHF gene generating at least one short isoform, often termed the “B” isoform (referred to as FGF13 in Singh et al.), and at least one isoform with a long N-terminal extension, referred to as the “A” isoform (named FGF13S in Singh et al.). FHFs are highly expressed in neurons and cardiac myocytes but can also be found in some nonexcitable tissues (12). Notably, they bind to and modulate the properties of all the VGSCs, except for perhaps Nav1.4, and are capable of modifying VGSC properties in complex ways. The most common impact of the binding of the FHF core to VGSCs is a depolarizing shift in the voltage dependence of inactivation and an enhancement of current density, both proexcitation effects. By contrast, A-type FHFs often induce a process known as long-term inactivation, where the channels appear to undergo normal fast inactivation but take much longer to recover than with typical, intrinsic VGSC fast inactivation (15). This long-term inactivation reduces action potential firing. Singh, Bernabucci, and authors focused on the B isoform of FGF13 (aka FHF2B) (11).

Genetic and preclinical studies have established FHFs as important physiological modulators of VGSCs (12). Point mutations in FGF12 and FGF13 (aka FHF1 and FHF2) cause epilepsy and intellectual disability (16, 17). Loss-of-function mutations in FHF14 are associated with spinocerebellar ataxia type 27 (18). While it is clear from these studies that FHFs contribute to epilepsy and ataxia, the role of FHFs in pain has been less clear. However, recently it was shown that FGF13 may be critical for heat nociception (19). Singh, Bernabucci, and authors (11) provide strong evidence that FGF13 can act as a pain rheostat in nociceptor neurons through its interactions with Nav1.7.

**Figure 1:**
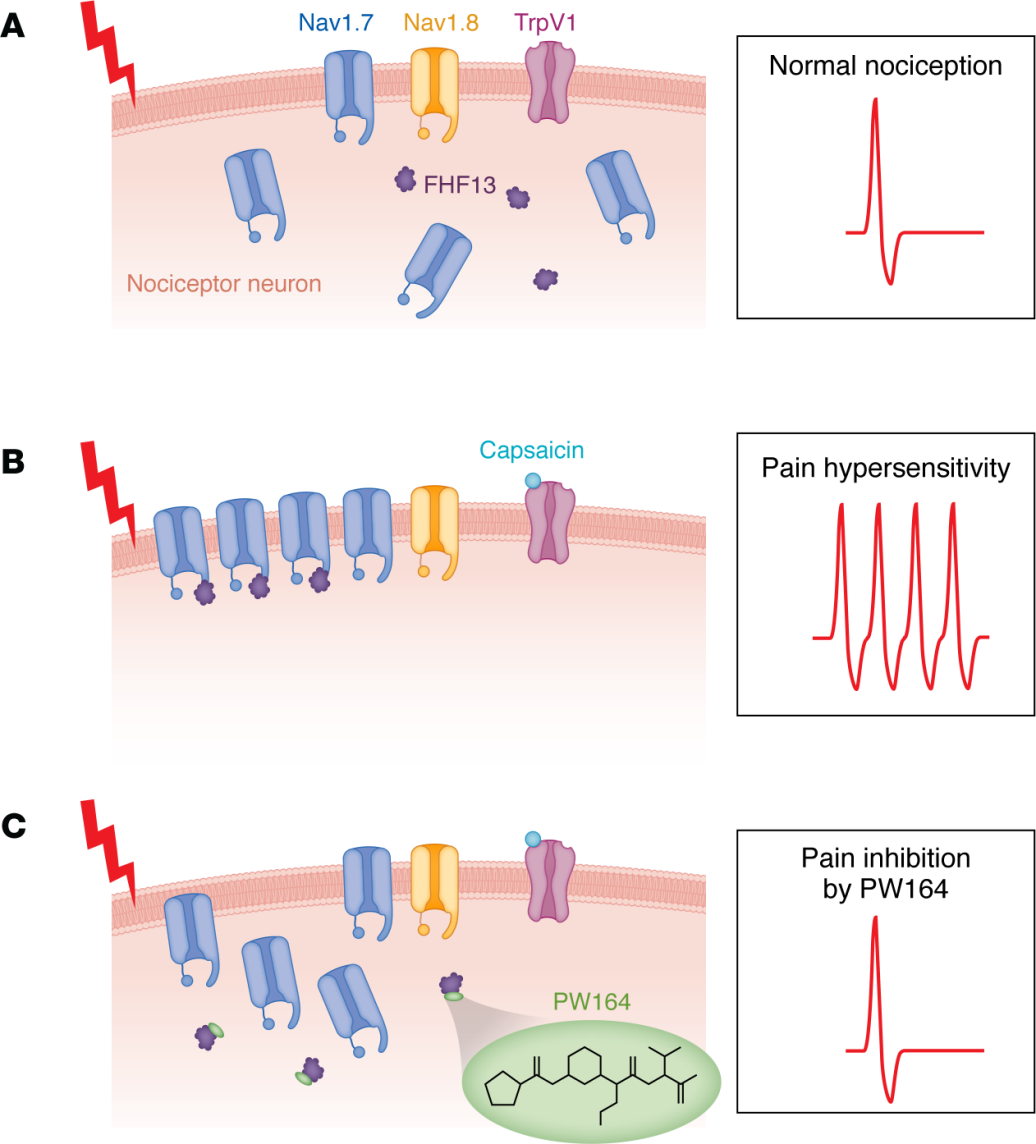
PW164 inhibits pain hypersensitivity. (**A**) Nav1.7 channels are expressed in nociceptor neurons along with FGF13 (FHF2B), Nav1.8, and the capsaicin receptor TrpV1. (**B**) Noxious stimuli and painful conditions enhance Nav1.7 surface expression and pain hypersensitivity by increasing the interaction between FGF13 and Nav1.7. (**C**) PW164 binding to FGF13 prevents the increase in Nav1.7 currents, inhibiting nociceptor activity and reducing pain.

Singh, Bernabucci, and colleagues used multiple techniques to determine how the FGF13 interaction with Nav1.7 modulates current properties, neuronal excitability, and pain sensations (11). Using protein-protein interaction assays, they identified a molecule with drug-like properties, PW164, that bound at the FGF13-Nav1.7 interface. PW164 inhibited FGF13 binding to Nav1.7 and blocked the ability of FGF13 to upregulate Nav1.7 current density (Figure 1). It also blocked capsaicin-induced sodium current in human induced pluripotent stem cell–derived sensory neurons without affecting baseline currents. This finding suggests a role for the FGF13-Nav1.7 complex in hypersensitivity following exposure to noxious stimuli. Indeed, in a mouse model where hypersensitivity was induced by paw injection of capsaicin, PW164 inhibited the capsaicin-induced thermal and mechanical hypersensitivity but not baseline thermal and mechanical responses. A compound that maintains baseline nociception provides an advantage, as normal pain is an important survival mechanism. To explore the role of the FGF13-Nav1.7 interaction in a more clinically relevant pain model, the authors tested PW164 in a mouse model of type-2 diabetic neuropathy (T2DN). Mechanical hypersensitivity was substantially reduced for several hours by injection of PW164, indicating that PW164-like compounds may be efficacious against at least some forms of clinically problematic pain. Additional screens also identified the small molecule ZL192 that (a) stabilized the FGF13-Nav1.7 interaction, (b) potentiated Nav1.7 currents when FGF13 was also present, and (c) induced robust mechanical hyperalgesia and thermal hypersensitivity in mice. This result further validated the importance of the FGF13-Nav1.7 interaction in regulating pain sensitivity.

## Unanswered questions and clinical implications

The study by Singh, Bernabucci, and authors (11) provides a compelling demonstration that FGF13 acts as a pain rheostat in nociceptor neurons and, importantly, further addresses some of the typical concerns with preclinical pain studies and targeting Nav1.7. To address the major question of whether cell and animal studies will translate to humans, the authors obtained human donor–derived sensory neurons from healthy patients and those with T2DN. Nav1.7 and FGF13 were coexpressed in human sensory neurons, with a higher degree of colocalization in neurons from patients with T2DN. Crucially, PW164 substantially reduced action potential firing elicited in the T2DN neurons. Hence, FGF13 regulates Nav1.7 currents in actual human neurons. Additionally, Singh, Bernabucci, and authors examined the impact of PW164 on blood pressure. They found no effect in mice, suggesting that PW164 does not have overt activity on the sympathetic nervous system. While the lack of effect on blood pressure in mice is encouraging, future studies will be critical to determine if manipulating the FGF13-Nav1.7 interaction in humans has impact on any aspects of sympathetic function in humans.

While Singh, Bernabucci, and authors provide an array of evidence that FGF13 is a critical player in modulating pain sensitivity (11), it is important to determine how selectively the FGF13-Nav1.7 interaction in nociceptive neurons can be targeted. Local anesthetics are effective at blocking pain sensations but are mainly administered to limited areas of the body to avoid systemic effects. Is PW164 specific enough in its actions and/or is it possible to generate more specific inhibitors of the FGF13-Nav1.7 interface? Surprisingly, PW164 bound FGF13-1b (FHF2B and FGF13U) but not the A isoform FGF13-1a (FHF2A and FGF13S) (11), suggesting isoform specificity, even though the core regions of both isoforms are fully conserved. However, it is not yet known if PW164 can interact with FHF3 or FHF4. Additionally, FGF13 can bind to most other VGSCs. PW164 binds FGF13 and blocks its interaction with Nav1.7 but, in Singh et al., PW164 did not alter Nav1.5 or Nav1.6 currents when these channels were coexpressed with FGF13 in HEK cells (11). This potential specificity is impressive. Regardless, it does not completely rule out the possibility that PW164 might have off-target effects. The c-terminal region of Nav1.7 that is thought to bind FGF13 is 94% conserved in Nav1.2, which was not tested. Also, other FGF13 targets may exist (12). Finally, although PW164 did not seem to have overt effects on sympathetic activity in the mouse studies (11), it is unclear if FGF13 modulates Nav1.7 currents in sympathetic neurons. Understanding the nuances in specificity may be crucial to pharmacological adjustment of the FGF13-Nav1.7 rheostat.

Overall, Singh et al. (11) highlights Nav1.7 as a viable target for development of analgesics, showing that protein-protein interactions that modulate VGSC activity provide a promising approach for the treatment of excitability disorders (Figure 1). It will be important to establish the full range of pain conditions that respond to disruption of the FGF13-Nav1.7 rheostat.

## Version 1 07/15/2025

Electronic publication

## References

[1] Coussens NP (2019). The opioid crisis and the future of addiction and pain therapeutics. J Pharmacol Exp Ther.

[2] Cummins TR (2007). The roles of sodium channels in nociception: Implications for mechanisms of pain. Pain.

[3] Wood JN (2025). Sensory neuron sodium channels as pain targets; from cocaine to Journavx (VX-548, suzetrigine). J Gen Physiol.

[4] Alsaloum M (2025). Voltage-gated sodium channels in excitable cells as drug targets. Nat Rev Drug Discov.

[5] Vasylyev DV (2024). Interplay of Nav1.8 and Nav1.7 channels drives neuronal hyperexcitability in neuropathic pain. J Gen Physiol.

[6] Yang Y (2018). Na_V_1.7 as a pharmacogenomic target for pain: moving toward precision medicine. Trends Pharmacol Sci.

[7] Woolf CJ (2020). Capturing novel non-opioid pain targets. Biol Psychiatry.

[8] Regan CP (2024). Autonomic dysfunction linked to inhibition of the Na_v_1.7 sodium channel. Circulation.

[9] Shin SM (2024). Peripherally targeted analgesia via AAV-mediated sensory neuron-specific inhibition of multiple pronociceptive sodium channels. J Clin Invest.

[10] Kanellopoulos AH (2018). Mapping protein interactions of sodium channel Na_V_1.7 using epitope-tagged gene-targeted mice. EMBO J.

[11] Singh AK (2025). Targeting the FGF13 rheostat in sensory neurons drives opposite nociceptive behaviors and provides a therapeutic strategy for diabetic neuropathy. J Clin Invest.

[12] Goldfarb M (2024). Fibroblast growth factor homologous factors: canonical and non-canonical mechanisms of action. J Physiol.

[13] Goldfarb M (2005). Fibroblast growth factor homologous factors: evolution, structure, and function. Cytokine Growth Factor Rev.

[14] Wang C (2012). Crystal structure of the ternary complex of a NaV C-terminal domain, a fibroblast growth factor homologous factor, and calmodulin. Structure.

[15] Dover K (2010). Long-term inactivation particle for voltage-gated sodium channels. J Physiol.

[16] Siekierska A (2016). Gain-of-function FHF1 mutation causes early-onset epileptic encephalopathy with cerebellar atrophy. Neurology.

[17] Fry AE (2021). Missense variants in the N-terminal domain of the A isoform of FHF2/FGF13 cause an X-linked developmental and epileptic encephalopathy. Am J Hum Genet.

[18] Laezza F (2007). The FGF14(F145S) mutation disrupts the interaction of FGF14 with voltage-gated Na^+^ channels and impairs neuronal excitability. J Neurosci.

[19] Marra C (2023). Enhanced sodium channel inactivation by temperature and FHF2 deficiency blocks heat nociception. Pain.

